# Interoception and Premonitory Urges in Children and Adolescents With Tic Disorders

**DOI:** 10.32872/cpe.8185

**Published:** 2023-03-31

**Authors:** Christina Schütteler, Katrin Woitecki, Manfred Döpfner, Alexander L. Gerlach

**Affiliations:** 1 Department of Psychology, Clinical Psychology and Psychotherapy, University of Cologne, Cologne, Germany; 2 School of Child and Adolescent Cognitive Behavior Therapy (AKiP), Faculty of Medicine and University Hospital Cologne, University of Cologne, Cologne, Germany; 3 Department of Child and Adolescent Psychiatry, Psychosomatics and Psychotherapy, Faculty of Medicine and University Hospital Cologne, University of Cologne, Cologne, Germany; Philipps-University of Marburg, Marburg, Germany

**Keywords:** heartbeat, muscle, EMG, interoceptive accuracy, predictive coding

## Abstract

**Background:**

Compared to healthy controls (HCs), adult Tic Disorder (TD) patients exhibit a lower interoceptive accuracy (IAcc) in heartbeat perception. Since the lower IAcc is not evident in children, the age at which tics develop, but in adults only (Pile et al., 2018, https://doi.org/10.1007/s10803-018-3608-8), lower IAcc may reflect a pathological mechanism relevant with regard to tics, premonitory urges (PUs) or the resulting impairment. Although tics are a motor phenomenon, up to date, IAcc has been assessed only with a heartbeat-counting task. This study aims at comparing cardiac and muscular IAcc using two different paradigms and investigates how IAcc is related to premonitory urges in youth.

**Method:**

Interoceptive measures (heartbeat-counting task, muscle tension paradigm) of 28 youth with TD were compared to 23 control participants and related to self-rated premonitory urges and tic symptoms.

**Results:**

TD patients did not differ from HCs in any IAcc measures. However, within TD patients, IAcc explained additional variance in PUs when controlling for tic severity. Muscular IAcc in TD patients is related to urges and tics, but the direction of this association is unclear. IAcc is lower in TD patients than in HCs, indicating imprecise sensory input which is more easily overcome by priors within the predictive coding framework.

**Conclusions:**

Muscle tension feedback tasks could extend interoceptive trainings aimed at improving IAcc to improve accuracy of urge perception (more precise sensory input) to foster the ability to control tics via HRT. Longitudinal studies could provide further insights in causal relationships between IAcc, premonitory urges and tics.

## Highlights

- A muscle tension paradigm assessed interoceptive accuracy.
- Patients with tics did not differ from healthy controls in interoceptive accuracy.
- Muscular interoceptive accuracy in patients relates to premonitory urges and tics.
- Muscular tension feedback tasks could improve treatment via habit reversal training.

Tics are sudden repetitive movements or vocalizations that occur in up to 21% of children (Cubo et al., 2011; Kurlan et al., 2001). In most cases tics disappear with increasing age and remain stable in only about 1% of people worldwide (Robertson & Cavanna, 2008). Tics regularly are preceded by an unpleasant premonitory urge or sensation (PU). PUs are often perceived as an urge to move, an impulse to move, inner tension or restlessness and mostly occur in the face, neck, shoulders, arms or hands (Kwak et al., 2003).

In psychotherapy, the perception of PUs is both necessary and problematic. On the one hand, in line with habit reversal training, a precise perception of PUs improves the ability to successfully suppress tics (McGuire et al., 2015). On the other hand, PUs illicit tics, negatively reinforce tics and correlate with tic severity (Li et al., 2019).

The capability to perceive bodily signals (‘interoception’) entails several different facets: IAcc is defined as the process of accurately detecting and tracking internal bodily sensations (Garfinkel et al., 2015). Interoceptive sensibility refers to the self-reported attention given to and detection of interoceptive information. Finally, interoceptive awareness refer to the metacognitive correspondence between objective IAcc and self-report of interoceptive information (Garfinkel et al., 2015). In the following, we will only focus on IAcc based on the notion that IAcc may be an underlying dimension in PUs, necessary to perceive interoceptive sensations.

Adult individuals suffering from a TD exhibit a lower IAcc in a heartbeat perception tasks whilst reporting a heightened perception of sensory stimuli (interoceptive sensibility) as compared to individuals without tics. However, this lower IAcc is not evident in children but in adults only (Pile et al., 2018). So far, IAcc in individuals with TDs has exclusively been assessed with a heartbeat-counting task (Schandry, 1981). Arguably, tic symptoms are muscle movements. Thus, assessing IAcc by looking at the ability of an individual to perceive heart activity may not be the best test of the possible involvement of IAcc in TD. Consequently, this study plans to investigate whether IAcc in a muscle tension perception paradigm is associated with PUs in addition to or over the ability to perceive heart activity.

According to a predictive coding account of bodily symptom perception, bodily changes such as heart activity or muscle tension often are weak and imprecise signals. Against this background, a heightened interoceptive sensibility for tic related symptoms may be the result of overly precise interoceptive priors. Specifically, in TDs, an overactive putamen and insula may lead to overly precise predictions at hierarchically higher levels, overriding the actual weak and imprecise sensory inputs. The resulting prediction error may be reduced by performing an ‚involuntary’ tic and/or be the basis for the perception of an unpleasant PU (Rae et al., 2019).

On the behavioral level, PUs may represent a conditioned response to aversive external stimuli such as criticism, offenses or social marginalization as result of tic execution. In the course of a TD, tics may become associated with those negative emotional valences that subsequently constitute the PU. After a tic is executed, the unpleasant PU dissolves and ticking is negatively reinforced, promoting maintenance of tics (O’Connor, 2002). Following the O’Connor model, an attentional focus on PUs and tics may, over time, enhance the overly precise prior even further. As a result, unpleasant PUs are perceived even more, impairing patients’ quality of life. Indeed, adult TD patients with a long history of the experience of tics exhibit lower IAcc (Ganos et al., 2015; Rae et al., 2019). Simultaneously, in adults, physical sensations are self-reported more often (interoceptive sensibility) compared to individuals without tics (Rae et al., 2019). Since a lower IAcc is not evident in children but in adults only (Pile et al., 2018), lower IAcc in adults may reflect a failure to develop, over time, a better IAcc if individuals suffering from TD, which is commonly found in healthy individuals (Murphy et al., 2019). In consequence, given this overly precise prior, the experience of PUs continues and may even be strengthened into adulthood. However, given that IAcc, so far, has been assessed only with a heartbeat-counting task, it is important to additionally assess whether IAcc in children with TDs may be increased with regard to the perception of muscular activity, since muscles are involved in the execution of tics. We opted to assess facial muscle tension given that most TD patients experience at least one tic in the face (McGuire et al., 2016).

Thus, in this study we wanted to test the hypotheses, that children and adolescents with pathological tics exhibit lower IAcc with regard to both heart activity as well as facial muscle tone as compared to children and adolescents without tics. We also test the hypotheses, that variance in PUs is explained by IAcc scores. Furthermore, we compare muscular to cardiac IAcc, using two different paradigms.

## Method

### Participants

A total of 51 children and youth between 10 and 19 years old were recruited at the University Hospital Cologne (28 patients and 23 control participants) and surrounding schools. One patient fulfilled the criteria of a chronic TD, 27 patients fulfilled the criteria of Tourette’s Syndrome according to ICD-10. Inclusion criteria were a previously diagnosed tic disorder, age 10-21 years and fluency in German. Exclusion criteria were insufficient German language skills and the absence of any tic during the last week. Twenty of these 28 TD patients (71%) were male (13 of 23 HCs, 57%). 7 TD patients were diagnosed with a comorbid disorder via diagnostic checklists (5 ADHD, 1 OCD, 1 conduct disorder, 1 trichotillomania). Two TD patients received anti-tic medication (Aripiprazole, Tiapride), three received medication targeting ADHD (Methylphenidate). TD patients and HCs did not differ with respect to gender (χ^2^ = 1.229, *p* = .268) or age. CBCL total scores differed significantly between groups, but not YSR scores (Table 1).

### Procedure

A two-group design compared TD patients with control participants not suffering from a TD (HC). TD patients and their parents additionally completed questionnaires regarding tic symptomatology and other psychopathology measures. Participation took between 70 to 90 minutes. The experimental paradigms measuring IAcc were presented via computer screen. Participants received an allowance of 8€ per hour. The current study was carried out according to the Declaration of Helsinki. The Ethics Commission of the University of Cologne’s Faculty of Medicine approved the study (CSHF0044) and the study was pre-registered (see Supplementary Materials). All participants and their legal guardians gave informed consent. The data that support the findings of this study are openly available in figshare (see Supplementary Materials).

### Questionnaires

The German version of the Child Behavior Checklist (CBCL; Döpfner et al., 2014) is a caregiver report and assesses a variety of psychopathological symptoms. In the current study, the internal consistency of the total score was excellent (α = 0.93). The German version of the Youth Self Report (YSR; Döpfner et al., 2014) aims at children and youth, is constructed equivalently to the CBCL, and assesses self-reports of a variety of psychopathological symptoms. The total score exhibited good internal consistency in the current study (α = 0.84). Both CBCL and YSR consists of the subscales Aggressive Behavior, Anxious/Depressive Symptoms, Attention Problems, Rule-Breaking Behavior, Somatic Complaints, Social Problems, Thought Problems, and Withdrawal/Depression.

The self-rated Symptom Checklist for Tic Disorders SCL-TIC-S (SCL-TIC-S) and SCL-TIC-P (parent-rated) are part of the DISYPS-III diagnostic system (Döpfner & Görtz-Dorten, 2017). They each assess the number of tics. On a 5-point Likert-type scale for each tic the respective intensity (very mild to severe, irritates others), frequency (a few times a week to constantly, every few minutes), and overall impairment (very low, hardly disturbs to extreme) is assessed. Additionally, the SCL-TIC-S assesses overall controllability (very low to very high). A tic symptom score (range: 0 – 16) is calculated by multiplying intensity with frequency for each tic, summing up the results and dividing the sum by the number of tics (Döpfner & Görtz-Dorten, 2017). Internal consistency of the SCL-TIC total score in the current study was excellent (α = 0.91 for SCL-TIC-S, α = 0.92 for SCL-TIC-P).

The Premonitory Urge To Tic Scale (PUTS) consists of 10 items and assesses PUs (Woods et al., 2005). We used the German translation (Rössner et al., 2010). The 10th item asks about tic controllability and is usually excluded or interpreted separately to sustain internal consistency (Woods et al., 2005). PUTS’ internal consistency in the current study was acceptable (α = .75 for PUTS-9, α = .71 for PUTS-10).

### Experimental Measures

The Mental Tracking paradigm (Schandry, 1981) was employed to assess IAcc based on cardiac sensibility. Participants were instructed to concentrate on their heartbeats for three randomly presented time intervals (à 25s, 35s and 45s) and silently count the perceived heartbeats. They were instructed to only count heartbeats that they felt (Koch & Pollatos, 2014). After a trial run, ECG-electrodes assessed the participant’s ECG (sample rate: 512 Hz) and heart beats were assessed online using the software Uvariotest (Gerhard Mutz, Cologne, compare Meyerholz et al., 2019). A sound signal indicated begin and end of each time interval. Participants were not allowed to measure their pulse or time and were not informed about their average heart rate nor the length of each time interval. The Mental tracking paradigm is applicable for children and youth at least 10 years old (Koch & Pollatos, 2014). In the current study, internal consistency of the IAcc score for heart activity (HIAcc) based on the scores of the three time intervals was good (α = 0.94).

Facial EMG was assessed by skin electrodes placed on the masseter and corrugator supercilii on the left side of each participant’s face. EMG placement followed the EMG guidelines by (Fridlund & Cacioppo, 1986). Muscle tension IAcc was assessed with a paradigm originally developed by Flor et al. (1992). Reported muscle tension and EMG measures were correlated to form an IAcc score for the masseter (MIAcc) and corrugator supercilii (CIAcc), respectively. We opted for two facial muscles that have previously been used in muscle discrimination tasks, because most TD patients display facial tics (Flor et al., 1992). During the task, participants looked at a screen that represented their muscle tension as measured by EMG. Muscle tension was visualized by a soccer ball that moves along a line colored in red, green and yellow. Participants were instructed to keep the soccer ball in the green target zone for 2.5 seconds by tensing their facial muscles accordingly. A sound signal indicated the successful completion of the task. After regulating the respective muscle, participants reported the degree of muscle tension they believed the task required on a Likert-type scale ranging from 1 to 5. Since the axis of the colored line changed with the task (i.e., the green zone represented different intensities of muscle tension), participants needed to rely on interoceptive information only. Overall, participants went through 16 tasks as described above for each muscle. The required muscle tension levels varied in equal parts between 15%, 30%, 45% and 60% of the maximal achievable tension level for each participant. This maximal achievable tension level for each participant had been measured directly before the perception task by asking participants to tense the respective muscle as much as possible.

### Data Analysis

The correlation between perceived and via EMG measured muscle tension represent masseter and corrugator IAcc scores, respectively. The heartbeat perception accuracy score (HIAcc) indicated the ability to perceive one’s own heartbeat accurately and was calculated by employing the following formula with i = time intervals (25s, 35s, 45s) and no = measured heartbeats ns = counted heartbeats:


$$HIAcc=\frac{1}{3}\sum_{i=1}^{3}\left(1-\frac{\left|no_{i}-ns_{i}\right|}{no_{i}}\right)$$

The resulting scores ranged between 0 to 1 with higher scores indicating higher cardiac accuracy. Independent samples two-tailed *t*-tests or chi^2^-tests were used to compare clinical between group measures and group differences in IAcc scores. Effect sizes are indicated by Cohen’s *d*. All statistical tests are two-sided with *p* < .05. Pearson-product-moment-correlations and multiple regressions determined the relation between IAcc scores and tics or PUs, respectively. We used SPSS for these calculations.

## Results

### Interoception in Youth With Tic Disorders Does not Differ From Healthy Controls

When investigating IAcc scores, *N* = 2 masseter datasets, *N* = 5 corrugator datasets and *N* = 1 HIAcc dataset needed to be excluded due to technical failure. On average *M* = 13.18 corrugator trials (range from 2 to 16 trials) and *M* = 14.92 masseter trials (range from 10 to 16 trials) were valid and could be included.

Table 1 displays descriptive statistics for each group and group comparisons between TD patients and HCs. TD patients’ and HC’s masseter and corrugator IAcc scores are shown in Table 1. Masseter and corrugator IAcc scores did not significantly differ between TD patients and HCs. TD patients and HCs did not significantly differ in any of the three IAcc measures, even when TD patients with comorbidities were excluded and the analysis was repeated (HIAcc: *t* = -0.414, *df* = 49, *p* = .68; MIAcc: *t* = -1.795, *df* = 38, *p* = .08; CIAcc: *t* = -1.309, *df* = 40, *p* = .20).

**Table 1:** Group Differences

Variable	TD patients		Healthy Controls		*t* (*df*)	*p*	Cohen's *d*
	*n*	*M (SD)*	*n*	*M (SD)*			
Age	28	12.65 (2.21)	23	12.86 (2.47)	*t* = -0.324 (*df* = 49)	.75	-0.09
CBCL Total Score	27	32.19 (17.13)	21	11.24 (7.84)	*t* = 5.188 (*df* = 46)	< .001***	1.51
YSR Total Score	26	49.96 (18.50)	23	45.26 (10.62)	*t* = 1.072 (*df* = 47)	.29	0.31
Interoceptive Accuracy Scores							
HIAcc	27	0.59 (0.28)	23	0.61 (0.29)	*t* = -0.244 (*df* = 48)	.81	-0.07
MIAcc	26	0.42 (0.32)	23	0.53 (0.33)	*t* = -1.115 (*df* = 47)	.27	-0.32
CIAcc	26	0.26 (0.36)	20	0.43 (0.34)	*t* = -1.614 (*df* = 44)	.11	-0.48

*Note.* CBCL = Child Behavior Checklist; YSR = Youth Self Report; HIAcc = heartbeat perception accuracy score; MIAcc = masseter interoceptive accuracy score; CIAcc = corrugator interoceptive accuracy score.

***p < .001.

### IAcc in a Proprioceptive Perception Task Explains Variance in Premonitory Urges in Youth

SCL-TIC-S score was *M* = 5.54 (*SD* = 2.98, *N* = 26), mean SCL-TIC-P score was *M* = 6.22 (*SD* = 3.33, *N* = 27). The mean PUTS total score for 9 items was *M* = 18.14 (*SD* = 5.02, *N* = 28). CIAcc and MIAcc correlated substantially with each other indicating internal validity, while not correlating significantly with HIAcc. Table 2 gives an overview over correlations between interoception scores and tic symptoms including PUs.

**Table 2:** Pearson Correlations

Variable	1	2	3	4	5	6
1. PUTS-9						
*r*	–	.14	.47	-.06	.39	.24
*p*		.50	.01	.77	.05	.22
*n*		(27)	(26)	(26)	(26)	(27)
2. SCL-TIC-P						
*r*		–	.34	.06	.11	.00
*p*			.08	.77	.59	.99
*n*			(27)	(26)	(25)	(27)
3. SCL-TIC-S						
*r*			–	.12	.36	.07
*p*				.57	.08	.73
*n*				(26)	(25)	(27)
4. CIAcc						
*r*				–	.58**	-.30
*p*					.00	.14
*n*					(25)	(26)
5. MIAcc						
*r*					–	-.23
*p*						.27
*n*						(25)
6. HIAcc						–

*Note.* PUTS-9 = Premonitory Urge to Tic Scale (9 items); SCL-TIC-S = self-rated Symptom-Checklist for Tic Disorders; SCL-TIC-P = parent-rated Symptom-Checklist for Tic Disorders; CIAcc = corrugator interoceptive accuracy score; MIAcc = masseter interoceptive accuracy score; HIAcc = heartbeat perception accuracy score.

*p < .05. **p < .01.

In youth, tic symptoms vary substantially with age parallel to PUs, so tic severity should be accounted for when investigating PUs. Multiple regression analysis was used to investigate how IAcc relates to PUs. With PUTS total score as the dependent variable, self-reported tic total score was entered in Step 1 to control for tic severity. HIAcc and CIAcc and MIAcc scores were entered in Step 2. Adding the IAcc scores in Step 2 led only to a marginally significant change in *R*^2^. Note, however, that the MIAcc score explained significant variance in PUs in addition to tic severity in Model 2 (compare Table 3).

**Table 3:** Linear Model of Predictors of PUTS-9 Total Scores With Confidence Intervals Reported in Parentheses

Predictors	*b*	95% CI		*SE B*	β	*p*	Zero-order correlation	Partial correlation
		*LL*	*UL*					
Step 1								
Constant	13.45	9.39	17.50	1.96		< .001**		
SCL-TIC-S	0.78	0.13	1.42	0.31	.46	.02*	.462	.462
Step 2								
Constant	9.70	4.00	15.40	2.73		.002*		
SCL-TIC-S	0.50	-0.15	1.14	0.31	.30	.12	.462	.340
HIAcc	5.14	-1.51	11.79	3.19	.29	.12	.279	.339
CIAcc	-4.40	-10.62	1.82	2.98	-.31	.16	-.059	-.313
MIAcc	8.09	0.88	15.31	3.46	.52	.03*	.378	.464

*Note. R*^2^ = .21 (*p* = .020) for Step 1, Δ*R^2^* = .22 (*p* = .08) and *R* = .43 (*p* = .02) for Step 2. PUTS-9 = Premonitory Urge to Tic Scale (9 items); SCL-TIC-S = self-rated Symptom-Checklist for Tic Disorders; SCL-TIC-P = parent-rated Symptom-Checklist for Tic Disorders; CIAcc = corrugator interoceptive accuracy score; MIAcc = masseter interoceptive accuracy score; HIAcc = heartbeat perception accuracy score.

*p < .05. **p < .01.

## Discussion

In the present sample of children and adolescents with and without tics, we found that neither HIAcc nor proprioceptive IAcc scores differed between these groups. These results are in line with recent studies that also did not find any difference in interoceptive accuracy (IAcc) in children with TD (Pile et al., 2018). Our study extends those findings to the perception of muscle tension. Although IAcc scores were numerically lower in participants with TD, these differences did not reach significance (*d* = -.07 – .43). Whereas lower HIAcc compared to HCs has been established in adult TD patients, we were not able to demonstrate such differences in our sample of children and adolescents. However, such differences in IA may evolve with increasing age of the TD, equal to increasing duration of a childhood-onset TD and may be compared to a model of altered interoception in children with chronic pain: Top-down processes, such as expectations of uncontrollably ticcing, and bottom-up processes, such as a stressed bodily state, may lead to altered interoception over time (Hechler, 2021). It would consequently be highly interesting to assess a sample of adult individuals with TD using our muscle tension paradigm.

Within TD patients, recent studies found IAcc to be positively correlated with PUs in adults (Ganos et al., 2015; Rae et al., 2019). Our findings corroborate this assumption. Adding IAcc scores (MIAcc, CIAcc and HIAcc) to tic severity scores when predicting PUs, a substantial (Δ*R*^2^ = .22) additional amount of variance in PUs was explained. Note, that neither masseter nor corrugator IA scores were significantly correlated with HIAcc. Consequently, these different measures of interoceptive ability may cover different facets indicating that different body domains may matter when assessing IA. When taking a closer look at the results, the MIAcc (*r* = .39) correlated more strongly with the PUTS than the CIAcc (*r* = -.06), which even correlated negatively with the PUTS. In contrast to the corrugator supercilii, the masseter is more frequently contracted deliberately and thus might it be easier to control and it might be easier to estimate its tension. Arguably, this may result in a more reliable measure. However, the corrugator supercilii is linked with emotional expression (Tan et al., 2012) and, arguably, the location of the corrugator supercilii overlaps with locations most frequently affected by tics (McGuire et al., 2016). We therefore recommend that future studies nonetheless continue to assess both facial muscles when looking at IA with regard to muscular tension.

Herbert et al. (2012) compared interoceptive accuracy in eating disorders across two bodily domains – cardiac and gastric –and showed them to be inversely correlated. Similarly, we found cardiac and muscular IAcc to be inversely correlated in TD (*r*_CIAcc_ = -.30, *r*_MIAcc_ = -.23), although not reaching significance. Following the interpretation by Herbert et al., trying to control muscles that feel uncontrollably at times, might increase activation in the sympathetic nervous system which in turn might increase cardiac IAcc. In line with Flack et al. (2017), activation in the sympathetic nervous system might as well be the result of heightened levels of fear when focusing on muscles associated with unpleasant tic execution.

HIAcc score did marginally significantly correlate with the PUTS in our sample of children and adolescents. Children have smaller hearts and a lower stroke volume, associated with a higher heart rate which influences heartbeat detection positively (Knapp-Kline & Kline, 2005). In light of this it may be concluded that increased HIAcc promotes the perception of PUs in children.

In summary, these results on the association between IA and PU provide an ambiguous picture. On the one hand, IA clearly is associated with PU, but the direction of this association remains unclear. Following our hypotheses, we would have assumed that IA should be negatively associated with PU, as was the case for corrugator perception. In contrast, we find a clear positive association between the perception of cardiac activity as well as tension of the masseter and PU. Obviously, more research is needed here.

Note that the findings linking interoception and PUs rely on the PUTS to represent PUs. However, the PUTS is a self-report measure that more likely represents interoceptive sensibility. Interoceptive sensibility is known to be altered in children and adults with TD (Owens et al., 2011; Rae et al., 2019) and the measure is challenged by the usual problems associated with self-reports. The relative relationship of interoceptive accuracy, interoceptive sensibility und interoceptive awareness constitute PUs’ presence and cognitive, emotional and clinical consequences (Garfinkel et al., 2015).

We find it especially intriguing to interpret our findings within the predictive coding framework (Ainley et al., 2016; Friston, 2010; Khalsa et al., 2018; Rae et al., 2019). In this framework, a difference between sensory input and prior expectation results in a prediction error. Following Bayesian Inference, the prediction error may be resolved by updating the prior expectation or executing a movement to change sensory input. In TD patients, in hierarchical higher brain structures, an over-precise interoceptive prior might predict movements. If the weak bottom-up sensory input does not correspond with this prediction, the insula has to resolve the resulting interoceptive prediction error. The anterior insula is hypothesised to update predictions to reduce prediction errors (Seth, 2013; Seth et al., 2012) and is known to show functional abnormalities in TD patients. Due to the prior’s over-precision and weight, imprecise sensory input may be overcome by the prediction and the prediction error is being ‘explained away’ by the anterior insula as a premonitory sensation (urge to move). In adults, lower IAcc is found which, arguably, indeed indicates weaker priors. However, in line with previous research in adults (Ganos et al., 2015), we found that lower IA (heart activity and tension of the masseter) is correlated with lower PUs. Assuming that good IA, over time, leads to weaker priors, a smaller prediction error may result in less PU. In contrast, IA for the tension of the corrugator, was negatively correlated with PU. Following this finding, one could argue that the worse the perception of actual physical sensations in areas in which tics occur the more top-down predictions will overshadow interoceptive sensations and result in the perception of PUs (perceptual inference).

There are a number of limitations to the current study that offer opportunities for future research. Studies investigating the perception of muscle tension in adult TD patients are yet to be conducted to gain further insights on the development of PUs over the life span. Since the current cross-sectional study allows correlative interpretations only, the longitudinal comparison of chronological changes in HIAcc, MIAcc and CIAcc scores, PUTS and urge thermometers over the lifespan would provide further information on their etiological meanings. In addition, the exploratory findings on IA in child and adolescent TD patients need to be replicated, preferably in larger samples. Our study exclusively focused on IAcc in youth with TD. Future studies may extend our findings to interoceptive sensibility to disentangle the influence of interoceptive sensibility and IAcc on the self-reported perception on premonitory urges. Altogether, our sample size was relatively small, compromising statistical power to some degree.

TD patients usually differ from HCs not only with regard to tics but also with regard to comorbidities such as ADHD or OCD, depression or anxiety. When comparing TD patients to typically developing children, our study cannot account for the impact of comorbidities such as ADHD or OCD due to the relatively small sample size. Therefore, the relationship between interoception and tics and PUs in TD patients with multiple comorbid diagnoses is hard to disentangle. Panic and somatic ratings, for example, were found to correlate with higher HIAcc in adults in children (Eley et al., 2007). Comparing TD patients with a control group exhibiting matching comorbidities could help to disentangle the complex influences of comorbidities on IAcc and PUs. In line with Pile et al. (2018), additional instructions could be added to muscle tension perception tasks to reduce effects of inattention, a frequent comorbid symptom in TD patients, and task-misunderstanding.

The PUTS measures PUs as a whole but does not differentiate between context- and time-dependent urges as a state and the PU as a general trait. It is yet to be examined how context- and time-dependent urges, measured by urge thermometers, vary in relation with changes in PUs as a trait and changes in IAcc.

Similar to the PUTS that measures PUs as a general trait, the SCL-TIC-S and SCL-TIC-P measure self- and parent-reported mean tic severity over the course of a week. It is not clear how accurate self-assessed tic frequency reflects actual tic expression. On the one hand, both child and adult patients underestimate their tic expression (Müller-Vahl et al., 2014; Pappert et al., 2003). On the other hand, parent-reports cannot be accurate either. At least adolescent patients are not observed by their parents most of the day and tic frequency highly depends on context. Another study compared children’s self-reported account of tic frequency to objective video ratings of tic frequency in various experimental situations. Self-reported tic frequency related to objective measures depended on the situation. Interestingly, the higher children scored on the PUTS, the better their self-report predicted objective tic-frequency (Barnea et al., 2016). This implies that self-reported tic frequency depends on PUs and probably IAcc. Still, as our multiple regression analysis showed, IA explains variance in addition to self-reported tic frequency. The moderate correlation with parent-reported tic-frequency (*r* = .34) further validates self-reported tic-frequency.

The current study holds clinical implications. Interoceptive trainings specifically targeting interoceptive domains that are impaired in TD may be more beneficial than multisystem interventions (Khalsa et al., 2018), so research on the impact of interoceptive trainings aimed at improving heartbeat perception (Schaefer et al., 2014) could be expanded by adding muscle tension biofeedback tasks to improve accuracy of urge perception to foster the ability to control tics via HRT. Observations from intervention studies examining muscle tension biofeedback in TD would provide further insights in causal relationships between IAcc, PUs and tics.

## Supplementary Materials

The Supplementary Materials contain the following items (for access see Index of Supplementary Materials below):

- Pre-registration protocol
- Research data

SchüttelerC.
WoiteckiK.
DöpfnerM.
GerlachA. L.
 (2020). Supplementary materials to "Interoception and premonitory urges in children and adolescents with tic disorders"
[Pre-registration protocol]. PsychOpen. 10.17605/OSF.IO/V3ZKY
PMC1010315937065000

SchüttelerC.
WoiteckiK.
DöpfnerM.
GerlachA. L.
 (2021). Supplementary materials to "Interoception and premonitory urges in children and adolescents with tic disorders"
[Research data]. PsychOpen. 10.6084/m9.figshare.17121632
PMC1010315937065000

## Data Availability

For this article, a data set is freely available (Schütteler, Woitecki, Döpfner, & Gerlach, 2020).
